# Microemulsion-Based Polymer Gels with Ketoprofen and Menthol: Physicochemical Properties and Drug Release Studies

**DOI:** 10.3390/gels10070435

**Published:** 2024-06-29

**Authors:** Filip Otto, Anna Froelich

**Affiliations:** 1 Poznan University of Medical Sciences, Chair and Department of Pharmaceutical Technology, 3 Rokietnicka Street, 60-806 Poznań, Poland; f.otto@ump.edu.pl; 2 Poznan University of Medical Sciences, Chair and Department of Pharmaceutical Technology, 3D Printing Division, 3 Rokietnicka Street, 60-806 Poznań, Poland

**Keywords:** microemulsion, gel, polymer, ketoprofen, menthol, Franz diffusion cells, drug release

## Abstract

Ketoprofen is a non-steroidal, anti-inflammatory drug frequently incorporated in topical dosage forms which are an interesting alternatives for oral formulations. However, due to the physiological barrier function of skin, topical formulations may require some approaches to improve drug permeation across the skin. In this study, ketoprofen-loaded microemulsion-based gels with the addition of menthol, commonly known for absorption-enhancing activity in dermal products, were investigated. The main objective of this study was to analyze the physicochemical properties of the obtained gels in terms of topical application and to investigate the correlation between the gel composition and its mechanical properties and the drug release process. Microemulsion composition was selected with the use of a pseudoternary plot and the selected systems were tested for electrical conductivity, viscosity, pH, and particle diameter. The polymer gels obtained with Carbopol^®^ EZ-3 were subjected to rheological and textural studies, as well as the drug release experiment. The obtained results indicate that the presence of ketoprofen slightly decreased yield stress values. A stronger effect was exerted by menthol presence, even though it was independent of menthol concentration. A similar tendency was seen for hardness and adhesiveness, as tested in texture profile analysis. Sample cohesiveness and the drug release rate were independent of the gel composition.

## 1. Introduction

Non-steroidal anti-inflammatory drugs (NSAIDs) are widely applied in a number of medical conditions associated with pain, fever, and inflammation [1,2]. These active ingredients act by the inhibition of cyclooxygenase enzymes (COX-1 and COX-2), resulting in the reduction of prostaglandin synthesis. A significant area of NSAID application is musculoskeletal conditions affecting bones, joints, muscles, or larger areas including different tissues and anatomical structures [3]. The most common disorders comprise rheumatoid arthritis, osteoarthritis, low-back pain, neck pain, and gout, while the remaining musculoskeletal diseases are classified as other [4]. According to Global Burden of Disease, the data from 2019 indicate that about 1.71 billion people around the world are affected with different types of musculoskeletal problems, with a higher incidence in high-income countries [3]. It is noteworthy that these conditions may significantly contribute to a reduced quality of life. The affected patients may experience pain and limited mobility, leading to difficulties in performing daily activities. The mentioned conditions may be considered as a significant socioeconomic burden, being an important cause of sick leave and generating enormous costs related to the treatment, rehabilitation, and disability pensions [5,6]. The therapeutic approach to musculoskeletal pain should be multi-directional, involving exercises, physical therapies, pharmacological treatment, and surgical interventions, depending on the condition type and severity, as well as occurring comorbidities [7,8]. NSAIDs are among the most frequently applied drugs employed in the management of pain related to different musculoskeletal conditions. It is noteworthy that oral therapies are the most common; however, oral administration may not be suitable for all patients because of adverse gastrointestinal, renal, or cardiovascular effects [5,9]. In some cases, topical therapies can be considered as an alternative to oral treatment, particularly at the early stages of a disease when the symptoms can be classified as mild to moderate [10,11]. The available literature studies [12,13,14] indicate that topical NSAIDs can be equally effective as oral formulations in pain alleviation. Moreover, topical administration offers numerous advantages over the oral route including a reduction in the mentioned side effect risks, reduced risk of interactions with other drugs administered orally, and avoidance or reduction of hepatic first-pass metabolism [15].

**Figure 1:**
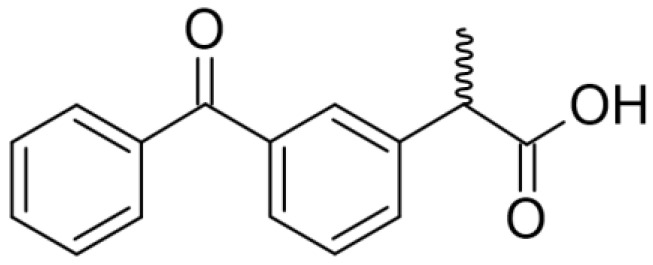
Chemical structure of ketoprofen (KET).

Ketoprofen (2-(3-benzoylphenyl)propionic acid; KET; Figure 1) is a drug with analgesic, anti-inflammatory, and antipyretic activity resulting from non-selective reversible inhibition of both cyclooxygenase 1 and 2 isoenzymes. It occurs in the form of a racemic mixture with the S-isomer displaying pharmacological activity, while the R-isomer is inactive. KET can be applied in numerous conditions with associated pain and inflammation, including musculoskeletal disorders, postoperative pain, and many others [16]. KET can be administered orally or parenterally. However, the systemic action of the drug is associated with an increased risk of side effects, including gastrointestinal problems. It is noteworthy that these adverse reactions can be avoided with topical formulations. KET, diclofenac, and ibuprofen are three popular NSAIDs applied externally and the existing evidence indicates that all of them can be employed in some less-severe conditions with good efficacy and minimized risk of adverse reactions [17].

One of the most important challenges related to the administration of pharmacologically active ingredients to the skin is its low permeability associated with its physiological barrier function. In order to overcome these difficulties, numerous strategies can be employed. One of the possible approaches frequently applied to increase the amount of the drug permeating across the *stratum corneum*, the most external skin layer playing a crucial role in preventing exogenous substances from entering the deeper tissues, is the selection of an appropriate drug carrier. Among the most extensively investigated ones, different types of nanocarriers, like submicron emulsions [18,19,20], vesicular systems [21], solid lipid and polymer nanoparticles [22,23], and drug nanocrystals [24], should be mentioned. Microemulsions are a type of submicron systems, composed of polar and non-polar phases stabilized with a surfactant and usually also a co-surfactant, which have an important role in the reduction of the interfacial tension between the phases to ultra-low values typical for these systems. Microemulsions are characterized by a spontaneous or low-energy formation process, which is an enormous advantage from a practical point of view. Numerous studies indicate that microemulsions can effectively enhance the permeation of the active ingredient across the *stratum corneum*, allowing for obtaining a better therapeutic effect [25,26,27,28,29]. Moreover, their composition, involving both polar and non-polar components stabilized with surfactants and low molecular weight co-surfactants revealing good solubilizing properties, allows for the incorporation of both lipophilic and hydrophilic compounds, comprising drugs and other components, like permeation enhancers [25,26]. It is also important to notice that microemulsions as low-viscosity media can be inconvenient for topical application and are frequently transformed into semisolid products, like polymer gels. It is noteworthy that mechanical characteristics of such complex systems, which also affect their sensory properties, depend on the applied gel and microemulsion composition [30,31,32]. 

Menthol (M) is a cyclic monoterpenoid alcohol occurring naturally in a volatile peppermint oil. It is frequently used as an additional active agent in topical pain relief products, as it displays some analgesic properties. The mechanism of its action is not clear. Menthol interacts with transient receptor potential melastatin-8 (TRPM8), which is responsible for the cooling sensation, and also acts as a weak sodium channel blocker and a vasodilating agent. It is noteworthy that these actions are observed at relatively low concentrations, while at higher ones (exceeding 30%), menthol interacts with heat-activated vanilloid receptors TRPV3 and reveals irritating properties [33,34,35]. Apart from its own activity observed after topical administration, menthol can be considered as a skin permeation enhancer, increasing the absorption of the main active ingredient and improving its efficacy [36,37,38]. 

In this study, the formulation studies and a characterization of novel microemulsion-based gels with ketoprofen as an active pharmaceutical ingredient are presented. As an additional analgesic and permeation-enhancing agent, menthol was applied. The main objective of this study was the evaluation of the physicochemical properties of the obtained systems with special attention paid to the impact of the gels’ composition on their mechanical features, including rheological and textural parameters important for dermal application. Finally, the analyzed formulations were tested for drug release with vertical Franz diffusion cells, in order to evaluate the correlation between the gel composition and ketoprofen diffusion rate essential for the therapeutic efficacy of the product. 

## 2. Results and Discussion

### 2.1. Microemulsion Preparation and Characterization

For microemulsion formulation, oleic acid, Brij^®^ O20, and ethanol were selected as the oil phase, surfactant, and co-surfactant, respectively. The choice of components was supported by the literature data regarding their application in dermal drug delivery, as well as the ability to form microemulsions. Oleic acid is commonly known for its activity as a skin absorption enhancer [39,40]. Moreover, in numerous studies it has been successfully employed as an oil phase in microemulsions [41,42,43,44]. It is noteworthy that it is a relatively polar oil, which is important in terms of water solubilization capacity. According to Rhee et al., the solubility of ketoprofen in oleic acid is higher compared to common oils, like olive, sesame, and corn oil, as well as mineral oil and isopropyl myristate [45]. Similar advantages have been described in the case of ethanol as a component of dermal microemulsions. Ethyl alcohol can be successfully employed both as a co-surfactant and a solubilizing agent [46,47,48]. Brij^®^ surfactants are popular non-ionic, surface-active agents employed in nano- and microemulsions [49,50], as well as liquid crystalline formulations [51] and solid lipid nanoparticles [52]. Brij^®^ O20, applied in this study, displays a relatively high HLB value of 15.5 [53], which can suggest high water solubilization capacity of the investigated system. 

**Figure 2:**
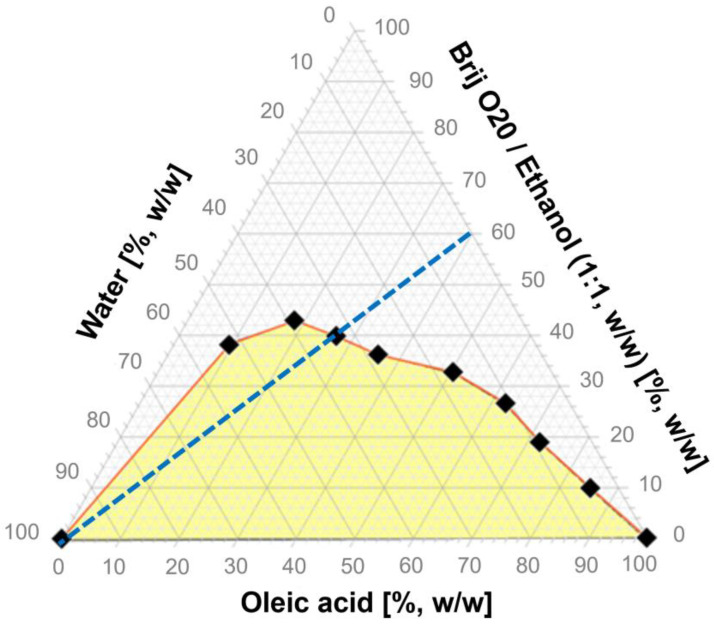
Pseudoternary plot obtained for the system composed of oleic acid, Brij^®^ O20/ethanol mixture, and water. The white area corresponds to the microemulsion systems. The blue dashed line depicts the dilution line followed in the conductivity study.

Pseudoternary phase diagrams are used to characterize the relationship between the system composition and its phase behavior. In microemulsion-related studies, they are employed to estimate the proportions between the polar and non-polar phase, as well as the surfactant and co-surfactant mixture, allowing for defining the domains indicating the occurrence of monophasic systems and other systems, like coarse emulsions. In Figure 2, a pseudoternary plot obtained for an oleic acid, water, and Brij^®^ O20/ethanol 1:1 (*w*/*w*) mixture is presented. The transparent area corresponds to clear isotropic liquids, while the yellow one corresponds to milky coarse emulsions. As the observed area is relatively high, which is related to the polarity of all of the applied components, no other surfactant/co-surfactant ratios were checked. For the further studies aiming at the recognition of particular microemulsion types, the system containing oleic acid and surfactant/co-surfactant mixture at 40:60 (*w*/*w*) ratio was selected. The selection criteria comprised the water incorporation capacity and surfactants content, which should be minimized whenever dermal delivery is taken into consideration. The selected system contained an intermediate amount of Brij^®^ O20/ethanol 1:1 (*w*/*w*) mixture and displayed a relatively high ability to incorporate water. The system was diluted with 0.01% sodium chloride solution along the dilution line depicted in Figure 2 and the electrical conductivity was measured.

**Figure 3:**
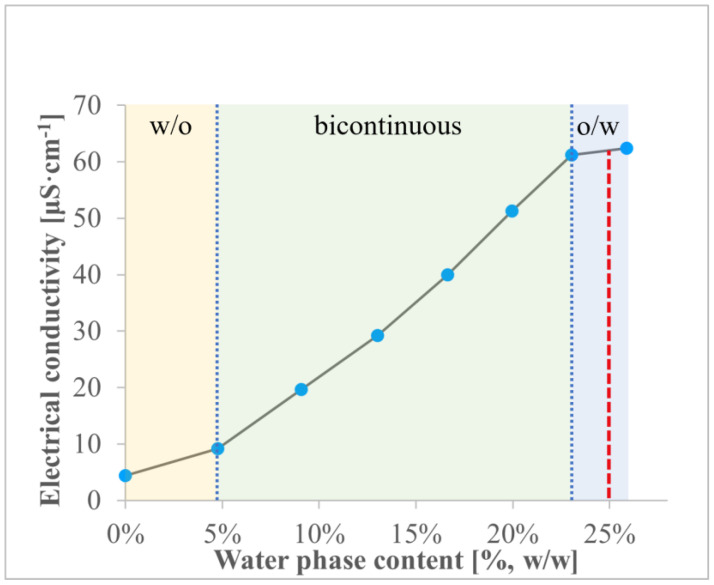
Electrical conductivity [μS cm^−1^] plotted as a function of water phase content [%, *w*/*w*] for the system containing oleic acid and surfactant/co-surfactant mixture at 40:60 (*w*/*w*) ratio. The red dashed line corresponds to the system selected for further investigations, while the blue dotted lines show the approximate structural transition points.

The plot depicting electrical conductivity vs. water phase content is presented in Figure 3. It is noteworthy that the electrical properties are associated with the microstructure of the system, and the structural transitions between the particular microemulsion types occurring with the increase in water phase content can be detected with the use of this method. In the first stage of the experiment, when polar phase content does not exceed 5%, the system reveals relatively low electrical conductivity, which is related to its structure. At this step, a w/o microemulsion is formed, with a continuous phase revealing low polarity and also a low ability to transport electrical charge. At about 5%, the electrical conductivity starts to increase, which is related to the structural transition to a bicontinuous microemulsion containing water channels in its structure. Further addition of the polar phase leads to an increase in the density of the channel network and a higher ability to conduct electrical charge. Finally, at about 23%, the system transforms into an o/w microemulsion and the polar channels merge into the continuous phase. Further water addition does not affect the conductivity, which stays approximately constant or might decrease if the polar phase is diluted. It must be emphasized that KET displays quite lipophilic properties and it may be expected that it also has a higher affinity towards oil phase than the water phase. In order to obtain a microemulsion system with the drug incorporated in nanosized internal phase droplets, an o/w system was taken into consideration. For further analyses, a microemulsion composed of 30% of oleic acid, 45% of Brij^®^ O20/ethanol (1:1, *w*/*w*), and 25% of water was used. 

The selected microemulsion system was used for the incorporation of KET and M. The obtained systems were transparent and no phase separation was observed upon the addition of the drug and menthol. For all obtained microemulsions, pH and the dynamic viscosity were measured and the dynamic light scattering (DLS) experiments (See Supplementary Materials) were performed to check the droplet diameter and system polydispersity. The results of these studies are summarized in Table 1 and Table 2.

The measured pH values were similar for all investigated microemulsions systems. All microemulsions revealed Newtonian properties, with constant viscosity values over the full shear rate range, which is typical for these systems [54]. In all investigated samples, the viscosity values were approximately 30 mPa s. 

The results of the DLS studies performed for the microemulsions indicate the presence of particles with a diameter within the nanometric range. It is noteworthy that the main peak in the case of the placebo system was located at a higher value, while in KET-loaded systems it was approximately 2 nm. The obtained results may indicate the localization of the drug molecules in the interfacial layer. However, it must be emphasized that microemulsions are concentrated systems susceptible to multiple scattering effects and cannot be diluted without significant structural alterations. As was already indicated by other authors, without proper corrections, the results of these studies should be interpreted cautiously and usually some corrections should be made [55,56,57,58]. 

### 2.2. Microemulsion-Based Gels: Preparation and Characterization

The compositions of microemulsion-based gels with their pH values are presented in Table 3. As a thickening agent necessary to obtain semisolid systems, Carbopol^®^ EZ-3 was used with the addition of the neutralizing agent (triisopropanolamine, TIPA). Carbopol^®^ is one of the most commonly applied pharmaceutical excipients, frequently employed to obtain hydrogels, hydroalcoholic gels, and complex systems [59,60,61]. The polymer has been known since 1950s and, therefore, it is well characterized and can be safely used in topical pharmaceutical products [62,63]. TIPA was selected over other neutralizing agents because of its high compatibility with alcohol-loaded systems [64]. The obtained gels were further analyzed for their mechanical properties, including rheological and textural parameters, as well as drug release. 

#### 2.2.1. Rheological Studies

**Figure 4:**
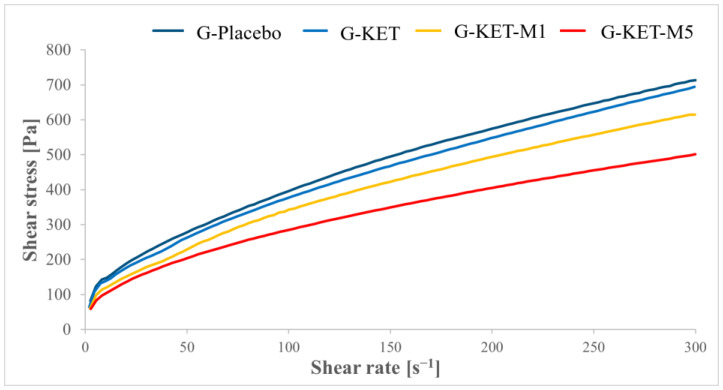
Flow curves obtained for the analyzed microemulsion-based gels in controlled shear rate mode.

**Figure 5:**
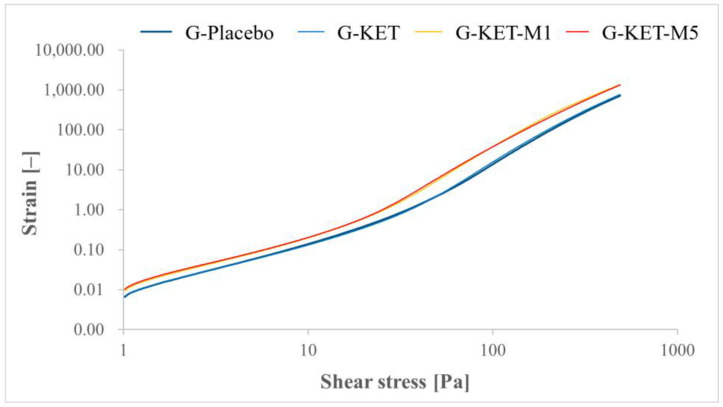
Flow curves obtained for the analyzed microemulsion-based gels in controlled stress mode.

**Figure 6:**
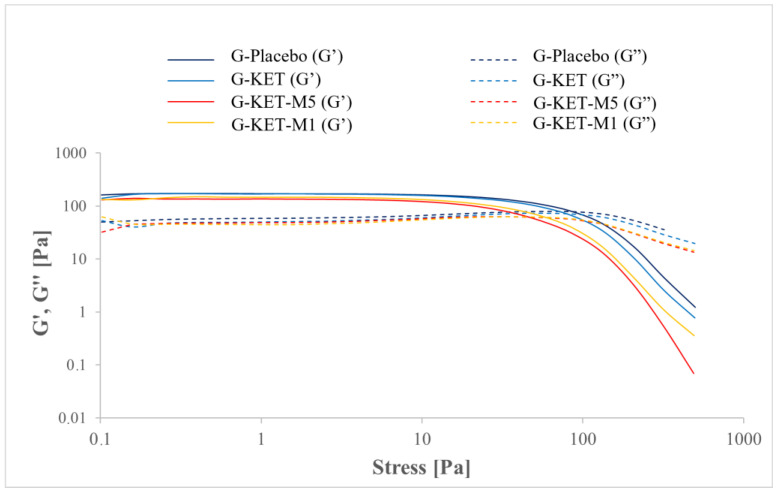
Storage (G′) and loss (G″) moduli plotted as a function of oscillatory stress in stress sweep studies.

**Figure 7:**
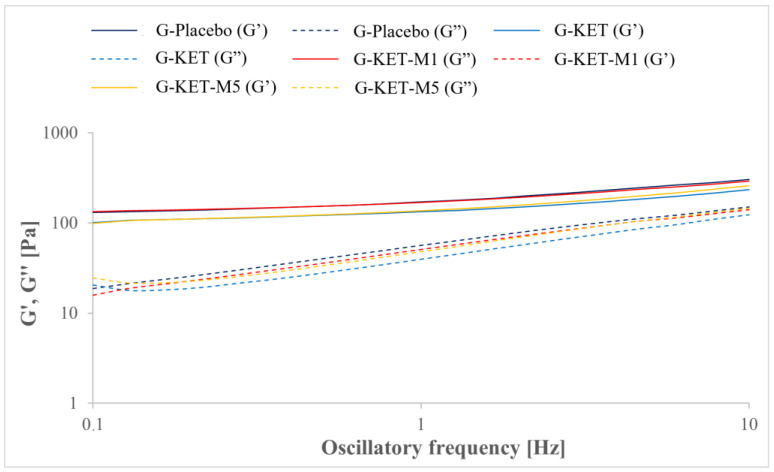
Storage (G′) and loss (G″) moduli plotted as a function of oscillatory frequency in frequency sweep studies.

Rheological tests comprised steady shear experiments and oscillatory studies, aiming at the description of viscoelastic properties. The flow curves obtained in controlled shear rate (CR) and controlled stress (CS) modes are presented in Figure 4 and Figure 5, respectively. The results of the tests conducted in CR mode were fitted to Herschel–Bulkley model (Equation (1)) [65]:

(1) $$\tau =\tau_{0}+K{\dot{\gamma }}^{n}$$

where τ is the shear stress, $\tau_{0}$ is the yield stress, K is the consistency factor, $\dot{\gamma }$ is the shear rate, and n is the power law index. In the case of CS studies, yield points were calculated as the intersection points of the two tangential lines fitted to the approximately linear parts of the curve. The plots obtained in oscillatory stress sweep (SS) and oscillatory frequency sweep (FS) tests are presented in Figure 6 and Figure 7, respectively. The curves obtained in SS mode were used to analyze the crossover points of G′ = f(τ) and G″ = f(τ) curves. All the calculated parameters are summarized in Table 4.

According to the obtained results, the analyzed formulations revealed non-Newtonian shear-thinning behavior, which is typical for Carbopol^®^-based semisolid gels [66,67]. Due to shear stress forces, hydrogen bonds and other weak interactions between the polymer chains forming a three-dimensional gel structure are broken. The gradual alignment of the polymer chains along the flow direction results in a decrease in viscosity as the shear rate increases. The observed behavior in dermal products designed for spreading on the skin surface can be generally considered as advantageous, as the application is more comfortable due to the viscosity drop upon rubbing [68]. Shear-thinning properties in all investigated systems are reflected by the n values calculated in Herschel–Bulkley model, which do not exceed 1 (Table 4). It is noteworthy that the n values recorded for the analyzed gels are similar, as well as the consistency indices. In the case of yield stress points obtained in both CR and CS mode, the highest values were observed for placebo gel. The presence of KET slightly decreased the yield stress values and the effect was enhanced in the presence of M. The difference between the formulations containing 1 and 5% M was not significant. 

In oscillatory stress sweep (SS) studies, storage and loss moduli (G′ and G″, respectively) were analyzed as a function of oscillatory amplitude. In all investigated samples, the initial prevalence of G′ over G″ values was seen (Figure 6). It is noteworthy that the increase in oscillatory amplitude eventually led to the intersection of both curves. The oscillatory stress values at the crossover points are summarized in Table 4. It is noteworthy that in the case of placebo and KET-loaded gel the values are similar, while in the presence of M a significant decrease is observed, which is similar to the yield stress points obtained in CR and CS modes. Stress sweep studies were also used to the estimate linear viscoelasticity range (LVR), which was necessary to select the stress value of 1 Pa for frequency sweep (FS) tests (Figure 7). The results of FS studies also indicate the prevalence of elastic properties over the viscous ones. According to the classification of gels provided by Clark and Ross-Murphy [69,70], the investigated systems can be classified as weak physical gels, as G′ is only slightly higher than G″ and both parameters depend only slightly on the frequency. A similar behavior has already been described for Carbopol^®^-based hydrogels [71] and other microemulsion-based gels [72]. 

#### 2.2.2. Texture Profile Analysis (TPA)

Textural studies have been extensively used in the food industry for the characterization of various food products and the description of parameters affecting their sensory properties [73,74,75]. It is noteworthy that these analyses can also be equally useful in other scientific and industrial areas requiring mechanical characterization of different objects. In pharmaceutical technology and personal care product development, texture profile analysis can be an important tool for providing valuable information complementary to the data obtained with rheological methods, as has already been mentioned by other authors [76,77]. 

**Figure 8:**
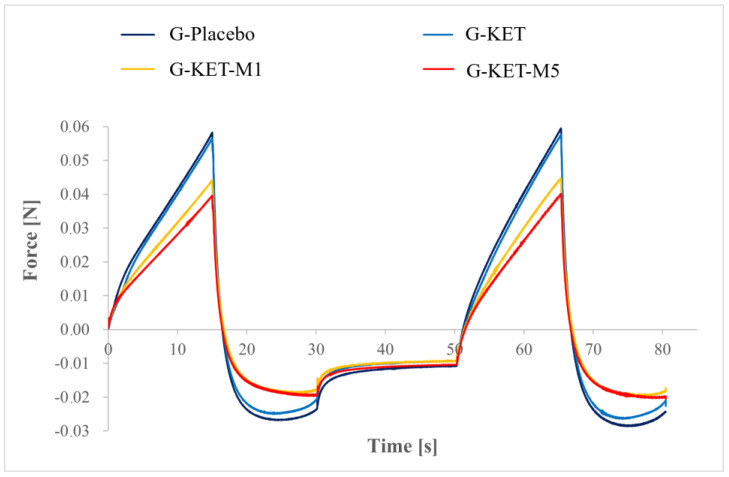
Texture profiles obtained for the gels investigated in this study.

The plots depicting the relationship of the measured force and time in texture profile analysis are presented in Figure 8, while the textural parameters calculated for the gel samples are summarized in Table 5. The highest hardness was observed for placebo sample; however, the results obtained for KET-loaded gel without menthol were similar. Significantly lower hardness values were observed for M-loaded samples and it should be noted that the parameters also depended on menthol concentration. The highest adhesiveness values were also observed for placebo samples and slightly lower ones were observed for G-KET samples. Significantly lower values were presented by M-loaded gels and the effect was independent of M concentration. The lower hardness and adhesiveness observed in the case of M-loaded gels can be correlated with the significantly lower yield stress values measured in rheological studies. Cohesiveness of the analyzed gels seems to be independent of their composition, as similar values were recorded for all of them.

#### 2.2.3. The Drug Release Studies

**Figure 9:**
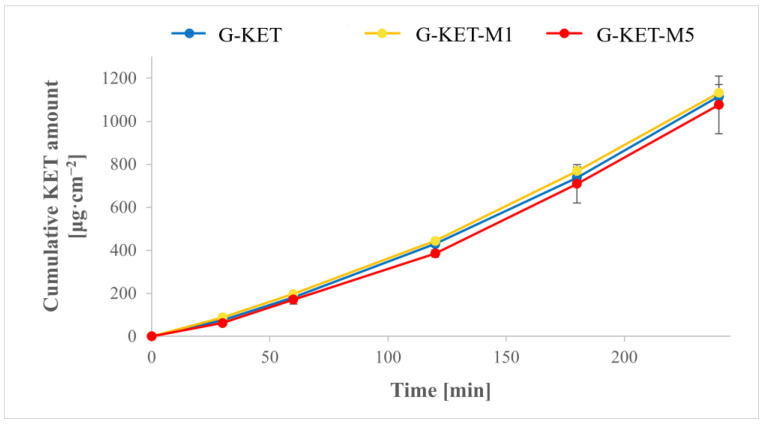
Cumulative amount of KET released over 4 h.

The cumulative amounts of KET released from the investigated gels plotted over time are depicted in Figure 9. The obtained results indicate that all of the investigated gels displayed the same behavior and the drug release process was independent of the sample composition and mechanical features. It is noteworthy that in the case of menthol-loaded gels significantly lower yield stress, hardness, and adhesiveness values were observed. Moreover, in CR and CS rheological studies, generally lower viscosities were recorded for these systems. As has already been mentioned in numerous literature reports [78,79,80,81,82], viscosity is crucial for drug release and, usually, lower diffusion rates are observed in the case of higher-viscosity media. However, in this study, no such correlation was found. 

Menthol, incorporated in the investigated samples as a potential skin permeation enhancer, did not affect the diffusion rate either. However, it must be emphasized that penetration enhancers interact with the skin structures, temporarily disturbing the ordered lipid chains in the *stratum corneum*, thereby increasing its permeability. In this study, a porous regenerated cellulose membrane was used in order to observe the potential effect of the formulation composition on KET diffusion and to eliminate any additional effects related to skin physiology. However, it should be emphasized that an experimental setup involving either ex vivo skin samples or in vivo conditions might give different results related to a potential interaction between the formulation components and the structures present in the *stratum corneum* [37]. 

## 3. Conclusions

In this study, an o/w microemulsion with ketoprofen was obtained and transformed into semisolid Carbopol^®^-based gels with or without menthol. The rheological studies revealed that all of the obtained gels had non-Newtonian, shear thinning behavior, typical for Carbopol^®^-thickened systems. The highest yield stress points measured with different procedures were observed in the case of placebo samples. The incorporation of ketoprofen decreased the yield points slightly and a stronger drop was seen as a result of menthol presence. Based on the results of the oscillatory frequency sweep, the analyzed samples were classified as weak physical gels. The texture profile analysis revealed that hardness and adhesiveness were the highest for the placebo gel, followed by the ketoprofen-loaded system without menthol; the lowest values were seen for gels containing both ketoprofen and menthol. Cohesiveness was approximately the same for all analyzed systems. Drug release studies showed no differences regarding the drug diffusion rate across the membrane, which means that neither the presence of menthol nor the differences in the rheological and textural properties affected the ability of the investigated gels to release ketoprofen. 

## 4. Materials and Methods

### 4.1. Materials

Brij^®^ O20 (Croda; Snaith, UK) and Carbopol^®^ EZ-3 (Lubrizol, Wickliffe, OH) were kindly provided free of charge by Croda Poland sp. z o.o. and Lubrizol, respectively. Oleic acid, triisopropanolamine (TIPA), and phosphate-buffered saline (PBS) tablets (pH = 7.4) were purchased from Sigma-Aldrich (Saint Louis, MO, USA) and used as received. Ethyl alcohol (99.8%), HPLC-grade acetonitrile, acetic acid (99.5%), and sodium chloride were purchased from Avantor™ Performance Materials Poland S.A. (Gliwice, Poland). Ketoprofen was purchased from Glentham Life Sciences^®^ (Corsham, UK). Menthol was purchased from Fagron (Rotterdam, The Netherlands) and potassium dihydrogen phosphate was obtained from Merck Millipore (Burlington, MA, USA). In all experiments, ultrapure water was used. 

### 4.2. Methods

#### 4.2.1. Microemulsion Preparation and Characterization

The pseudoternary phase diagram was prepared with a water titration method at 25.0 ± 0.5 °C. In the first step, Brij^®^ O20/ethanol mixture (1:1, *w*/*w*) was prepared. Next, 4.0 g samples with oil and surfactant mixtures at different weight ratios (1:9, 2:8, 3:7, 4:6, 5:5, 6:4, 7:3, 8:2, and 9:1) were prepared and titrated with ultrapure water until turbidity occurred. During the titration, the samples were gently mixed and inspected visually. The compositions corresponding to the transparent monophasic liquids were classified as microemulsions and the turbid systems were assigned to the coarse emulsion region. The obtained results were plotted with the use of https://www.ternaryplot.com/ (accessed on 23 April 2024).

The electrical conductivity studies were performed at 25.0 ± 0.5 °C with FiveEasy™ conductivity meter (FE30; Mettler Toledo, Greifensee, Switzerland) calibrated with 1413 μS/cm and 12.88 mS/cm conductivity standards. All measurements were performed in triplicate and average values with standard deviations were calculated. The analyzed system (20.0 g) was composed of oleic acid and Brij^®^ O20/ethanol mixture at a 40:60 ratio (*w*/*w*). The system was titrated with 0.01% sodium chloride solution; after the addition of each portion (1.0 mL), it was gently mixed and the conductivity was measured. 

The microemulsions selected for further studies were prepared by mixing oleic acid with Brij^®^ O20/ethanol mixture, dissolving solid components (i.e., ketoprofen and menthol) in the resulting solution and adding water in the last step. The mixture was gently stirred until transparent, monophasic liquid was obtained. 

The microemulsions were tested for pH with the use of CG 842 Schott pH-meter (Schott Instruments GmbH, Weilheim, Germany) equipped with SenTix^®^ Sp-DIN probe (WTW, Pomiarowy i Analityczny Sprzęt Techniczny Sp. z o. o., Wrocław, Polska). All measurements were performed in triplicate and average values were calculated. 

The viscosity of microemulsions was measured with a rotational rheometer HAAKE™ Rheostress1 (Thermo Electron Corp., Waltham, MA, USA) equipped with a Thermo HAAKE™ DC 30 temperature-controlled unit and coaxial cylinders Z20 DIN (sample volume: 8.2 mL, measurement gap: 4.2 mm). The tests were performed in triplicate at 25.0 ± 0.5 °C and average viscosity values were calculated. The analyses were performed in controlled shear rate mode, with shear rate increasing linearly from 1 to 200 s^−1^. The obtained $\tau =f\left(\dot{\gamma }\right)$ relationship was fitted to Newton model. 

The particle size was measured at 25.0 ± 0.5 °C with the use of the dynamic light scattering (DLS) method, employing Zetasizer Nano ZS (Malvern Instruments Ltd., Worcestershire, UK) equipped with a He-Ne laser light source (λ = 633 nm) operating in a backscattering mode (detection angle: 173°). Approximately 1 mL of an undiluted sample was placed in a disposable cuvette and sealed with parafilm to avoid evaporation. Prior to the DLS experiment, refractive indices of the samples were measured with a digital handheld refractometer DR201-95 (Kruss Optronic™, Hamburg, Germany). 

#### 4.2.2. Microemulsion-Based Gels: Preparation and Characterization

In the first step of the gel preparation procedure, KET and M were dissolved in the mixture containing oleic acid and Brij^®^ O20/ethanol 1:1 mixture. Next, water was added and the system was gently mixed. In the following step, Carbopol^®^ EZ-3 was dispersed with the use of an overhead stirrer at 1000 rpm. After the uniform mixture was obtained, 50% TIPA solution was added and the resulting gel was mixed further at 500–600 rpm. 

##### Rheological Studies

All rheological analyses were performed with a rotational rheometer HAAKE™ Rheostress1 (Thermo Electron Corp., Waltham, MA, USA) equipped with Thermo HAAKE™ DC 30 temperature-controlled unit and parallel plates (PP35 Ti; measurement gap: 1.000 mm). All tests were conducted at 25.0 ± 0.5 °C in triplicate with a fresh gel portion used in each run and average values were calculated. In rheological studies, the following test types were performed:

- Flow behavior study in controlled shear rate mode (CR; shear rate: 1.0–300.0 s^−1^, measurement time: 60 s);
- Flow behavior study in controlled shear stress mode (CS; shear stress: 1.0–500.0 Pa, measurement time: 60 s);
- Oscillatory stress sweep (SS; oscillatory stress: 0.1–500.0 Pa; frequency was kept constant at 1.0 Hz);
- Oscillatory frequency sweep (FS; oscillatory frequency: 0.1–10.0 Hz; stress was kept constant at 1.0 Pa, which was based on the results of SS tests).

##### Texture Profile Analysis (TPA)

This study was conducted with the use of Autograph AGS-X texture analyzer (Shimadzu, Kyoto, Japan). Approximately 20 mL of gel samples were placed in 25 mL beakers. The measurements were performed in triplicate at an ambient temperature. The sample was compressed twice with a steel cylindrical probe (diameter: 20 mm) moving at 60 mm min^−1^ velocity to the depth of 15 mm. The interval time between the compression cycles was 20 s. The obtained results were presented as a force vs. time relationship. For all gel samples, hardness, adhesiveness, and cohesiveness were determined [83].

##### Drug Release Studies

Ketoprofen release was investigated with the use of vertical Franz diffusion cells (PermeGear, Hellertown, PA, USA). The acceptor compartments of the cells were filled with PBS (pH = 7.4) solution, while in the donor compartments approximately 1.0 g of gel was placed. Between donor and acceptor compartments, regenerated cellulose SnakeSkin™ membranes (molecular weight cutoff: 10 kDa) (Thermo Scientific™, Waltham, MA, USA) were mounted. The effective diffusion area was 0.999 cm^2^. The acceptor fluid during the experiment was stirred at 200 rpm and the temperature was set at 32.0 ± 0.5 °C. The samples (0.2 mL) were withdrawn at 30, 60, 120, 180, and 240 min timepoints and immediately replaced with an equal amount of fresh PBS. 

The drug concentration in the collected samples was determined with a validated high-performance liquid chromatography (HPLC) method. For the analyses, a UHPLC Nexera-i LC-2040C system (Shimadzu, Kyoto, Japan) equipped with a C18 Superspher, Mz-Analysentechnik column (250 × 4 mm, 4 μm) was used. The analyses were performed under isocratic conditions with the mobile phase consisting of acetonitrile and potassium dihydrogen phosphate solution (2.72 g L^−1^) adjusted to pH = 2.5 with acetic acid (60:40, *v*/*v*; flow rate: 1.0 mL min^−1^). The analytical wavelength was 255 nm, the column temperature was set at 30.0 °C, and the injection volume was 50 μL. The limit of detection for the validated HPLC method was 12.16 μg·mL^−1^, while the limit of quantification was 36.84 μg·mL^−1^.

Based on the determined drug concentrations, the cumulative drug amount that diffused in each timepoint to the acceptor compartment was calculated with the use of Equation (2) [84]:

(2) $$Q=\frac{C_{n}\cdot V+\sum_{i=1}^{n-1}C_{i}\cdot S}{A}$$

where *Q* is the cumulative drug amount, *C_n_* is the KET concentration determined at the nth sampling point, *V* is the Franz cell volume, $\sum_{i=1}^{n-1}C_{i}$ is the sum of the drug concentrations determined at timepoints 1 through n-1, *S* is the withdrawn sample volume, and *A* is the effective diffusion area. 

## Figures and Tables

**Table 1:** Placebo, KET-loaded, and M-loaded microemulsion compositions and their pH and viscosity values.

	Microemulsions			
Component	Placebo	KET	KET-M1	KET-M5
KET [%, *w*/*w*]	-	2.5	2.5	2.5
M [%, *w*/*w*]	-	-	1.0	5.0
Microemulsion [%, *w*/*w*]	100.0	97.5	96.5	92.5
**pH**	4.89 ± 0.01	4.87 ± 0.01	4.82 ± 0.02	4.73 ± 0.01
**Viscosity** [mPa s]	31.29 ± 0.04	29.54 ± 0.02	29.34 ± 0.08	27.91 ± 0.03

**Table 2:** The results of DLS experiments performed for microemulsions.

Parameter	Placebo	KET	KET-M1	KET-M5
Peak size [nm]	3.279 ± 0.169	1.877 ± 0.022	1.916 ± 0.015	2.009 ± 0.029
Peak intensity [%]	73.2 ± 0.6	87.5 ± 1.0	90.5 ± 1.5	100.0 ± 0.0
Polydispersity index (PDI)	0.275 ± 0.001	0.266 ± 0.004	0.276 ± 0.003	0.264 ± 0.004

**Table 3:** The compositions and pH values measured for the microemulsion-based gels.

Component	G-Placebo	G-KET	G-KET-M1	G-KET-M5
KET [%, *w*/*w*]	-	2.5	2.5	2.5
M [%, *w*/*w*]	-	-	1.0	5.0
Carbopol^®^ EZ-3 [%, *w*/*w*]	2.0	2.0	2.0	2.0
TIPA [%, *w*/*w*]	0.2	0.2	0.2	0.2
Microemulsion [%, *w*/*w*]	97.8	95.3	94.3	92.5
pH	5.10 ± 0.02	4.81 ± 0.02	4.74 ± 0.01	4.71 ± 0.01

**Table 4:** Rheological parameters obtained as a result of CR and CS flow curve analysis.

Parameter	G-Placebo	G-KET	G-KET-M1	G-KET-M5
$\tau_{0}$ [Pa]	61.57 ± 2.81	56.92 ± 1.77	32.46 ± 1.81	29.64 ± 1.20
K [Pa s^n^]	21.77 ± 1.40	17.81 ± 0.61	19.19 ± 3.01	17.38 ± 3.18
n [-]	0.60 ± 0.01	0.63 ± 0.01	0.56 ± 0.02	0.61 ± 0.02
Yield point (CS) [Pa]	33.98 ± 1.45	29.67 ± 1.46	22.19 ± 2.18	18.68 ± 0.63
Crossover point (SS) [Pa]	84.00 ± 1.78	78.47 ± 2.87	60.29 ± 0.50	46.31 ± 2.29

**Table 5:** Textural parameters obtained in TPA test for gel samples.

Parameter	G-Placebo	G-KET	G-KET-M1	G-KET-M5
Hardness [mN]	58.37 ± 3.85	54.54 ± 2.76	44.25 ± 2.25	39.72 ± 1.12
Adhesiveness * [mJ]	568.9 ± 45.1	486.8 ± 31.9	433.28 ± 43.0	433.82 ± 75.0
Cohesiveness [-]	0.94 ± 0.01	0.98 ± 0.01	0.96 ± 0.01	0.97 ± 0.02

* Presented as absolute values.

## Data Availability

Dataset is available on request from the authors.

## Supplementary Materials

The following supporting information can be downloaded at: https://www.mdpi.com/article/10.3390/gels10070435/s1, Figure S1: DLS plot for placebo microemulsion; Figure S2: DLS plot for KET microemulsion; Figure S3: DLS plot for KET-M1 microemulsion; Figure S4: DLS plot for KET-M5 microemulsion; Figure S5: The DLS plot depicting particle size distribution in all investigated microemulsions. The red line corresponds to placebo sample, while the green one to KET, the blue one to KET-M1 and the black one to KET-M5.
