# Supplementary material for: Microemulsion-Based Polymer Gels with Ketoprofen and Menthol: Physicochemical Properties and Drug Release Studies
Source: Gels. 2024 Jun 29;10(7):435. doi: 10.3390/gels10070435 (PMC11275338; doi:10.3390/gels10070435)
Supplement: Supplementary file 1 [file gels-10-00435-s001.zip › gels-3038593-supplementary.pdf]

Supplementary materials

I. The results of the dynamic light scattering (DLS) experiments

The plots obtained in DLS experiments are depicted in Figures 1S-4S. The image depicting all of the obtained results together is presented in Figure 5S.

			Size (d.nm):	% Intensity:	St Dev (d.nm):
Z-Average (d.nm):	0,6826	Peak 1:	3,279	73,2	0,9893
PdI:	0,274	Peak 2:	0,6453	26,8	0,04220
Intercept:	0,854	Peak 3:	0,000	0,0	0,000
Result quality :	Good				

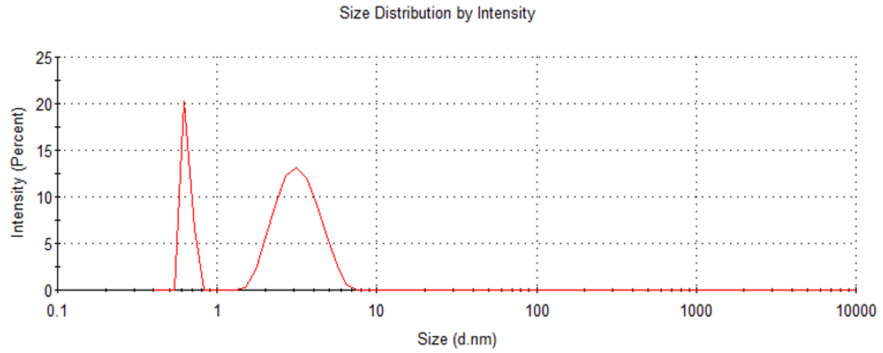

Fig. 1S. DLS plot for placebo microemulsion.

			Size (d.nm):	% Intensity:	St Dev (d.nm):
Z-Average (d.nm):	0,8418	Peak 1:	1,877	87,5	0,5355
PdI:	0,265	Peak 2:	0,6495	12,5	0,04939
Intercept:	0,899	Peak 3:	0,000	0,0	0,000
Result quality :	Good				

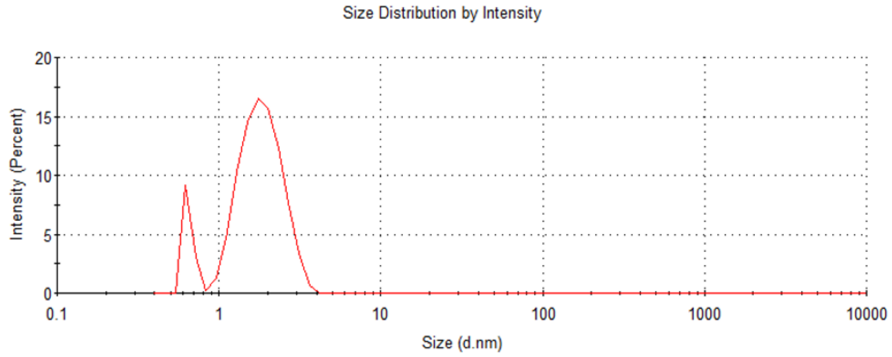

Fig 2S. DLS plot for KET microemulsion.

			Size (d.nm):	% Intensity:	St Dev (d.nm):
Z-Average (d.nm):	0,8689	Peak 1:	1,916	90,5	0,5450
PdI:	0,276	Peak 2:	0,6419	9,5	0,04004
Intercept:	0,891	Peak 3:	0,000	0,0	0,000
Result quality :	Good				

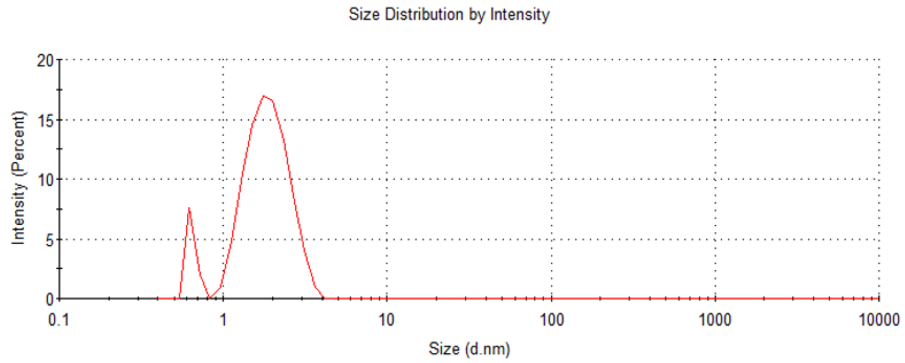

Fig. 3S. DLS plot for KET-M1 microemulsion.

			Size (d.nm):	% Intensity:	St Dev (d.nm):
Z-Average (d.nm):	1,294	Peak 1:	2,009	100,0	0,5988
PdI:	0,264	Peak 2:	0,000	0,0	0,000
Intercept:	0,886	Peak 3:	0,000	0,0	0,000
Result quality :	Good				

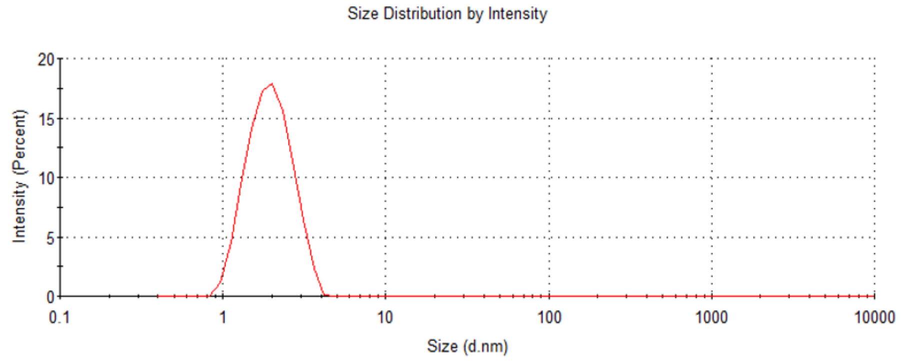

Fig. 4S. DLS plot for KET-M5 microemulsion.

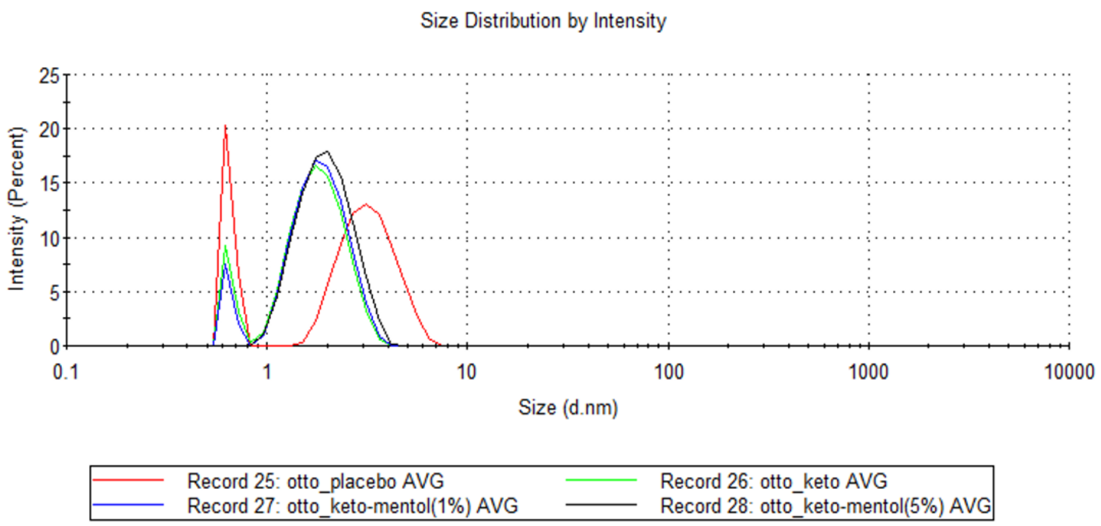

Figure 5S. The DLS plot depicting particle size distribution in all investigated microemulsions. The red line corresponds to placebo sample, while the green one to KET, the blue one to KET-M1 and the black one to KET-M5.
